# Examining vulnerability and resilience in maternal, newborn and child health through a gender lens in low-income and middle-income countries: a scoping review

**DOI:** 10.1136/bmjgh-2021-007426

**Published:** 2022-04-19

**Authors:** Fatima Abdulaziz Sule, Olalekan A Uthman, Emmanuel Olawale Olamijuwon, Nchelem Kokomma Ichegbo, Ifeanyi C Mgbachi, Babasola Okusanya, Olusesan Ayodeji Makinde

**Affiliations:** 1 Department of Research and Development, Viable Helpers Development Organization, Abuja, Federal Capital Territory, Nigeria; 2 Division of Health Sciences, University of Warwick, Coventry, UK; 3 Department of Obstetrics and Gynaecology, University of Lagos College of Medicine, Lagos, Nigeria; 4 Department of Research and Development, Viable Knowledge Masters, Gwarinpa, Federal Capital Territory, Nigeria

**Keywords:** child health, systematic review, health policy, maternal health, medical demography

## Abstract

**Introduction:**

Gender lens application is pertinent in addressing inequities that underlie morbidity and mortality in vulnerable populations, including mothers and children. While gender inequities may result in greater vulnerabilities for mothers and children, synthesising evidence on the constraints and opportunities is a step in accelerating reduction in poor outcomes and building resilience in individuals and across communities and health systems.

**Methods:**

We conducted a scoping review that examined vulnerability and resilience in maternal, newborn and child health (MNCH) through a gender lens to characterise gender roles, relationships and differences in maternal and child health. We conducted a comprehensive search of peer-reviewed and grey literature in popular scholarly databases, including PubMed, ScienceDirect, EBSCOhost and Google Scholar. We identified and analysed 17 published studies that met the inclusion criteria for key gendered themes in maternal and child health vulnerability and resilience in low-income and middle-income countries.

**Results:**

Six key gendered dimensions of vulnerability and resilience emerged from our analysis: (1) restricted maternal access to financial and economic resources; (2) limited economic contribution of women as a result of motherhood; (3) social norms, ideologies, beliefs and perceptions inhibiting women’s access to maternal healthcare services; (4) restricted maternal agency and contribution to reproductive decisions; (5) power dynamics and experience of intimate partner violence contributing to adverse health for women, children and their families; (6) partner emotional or affective support being crucial for maternal health and well-being prenatal and postnatal.

**Conclusion:**

This review highlights six domains that merit attention in addressing maternal and child health vulnerabilities. Recognising and understanding the gendered dynamics of vulnerability and resilience can help develop meaningful strategies that will guide the design and implementation of MNCH programmes in low-income and middle-income countries.

**What is already known on this topic?**

- Socioeconomic inequalities place women and girls in precarious positions that adversely affect their vulnerability and resilience to health shocks.
- Research on the gendered dimension of maternal and child health vulnerability and resilience is needed to fully evaluate how gender expectations may result in greater vulnerability for mothers, newborns and children or impact their resilience.

**What this study adds**

- This study provides new evidence on the gender dynamics of vulnerability and resilience in maternal, newborn and child health (MNCH) and how this impacts health outcomes.

**How this study might affect research, practice or policy**

- This research informs programme managers and researchers of the importance of embedding gender perspectives in future design of interventions aimed at addressing MNCH outcomes.
- It also calls for further research aimed at generating robust evidence on gender-transformative interventions that best address MNCH vulnerability and resilience across different contexts in low-income and middle-income countries.

## Introduction

Maternal and childhood mortality remains key health challenges in several low-income and middle-income countries (LMICs). In 2019, approximately 5.2 million children died before their fifth birthday, more than 80% of these deaths occurred in sub-Saharan Africa and Central and South Asia.^1^ Maternal mortality in sub-Saharan Africa and South Asia bore 86% of the estimated global burden in 2017.^2^ Sub-Saharan Africa’s maternal mortality ratio of 546 per 100 000 live births is estimated to be the highest globally for any region.^3^

In Maternal, Newborn and Child Health (MNCH), a vulnerable pregnant woman was defined as a woman who is threatened by physical, psychological, cognitive and/or social risk factors in combination with lack of adequate support and/or adequate coping skills.^4^ On the other hand, resilience has been described as the capability of the public health and healthcare systems, communities, and individuals to prevent, protect against, quickly respond to and recover from health emergencies, particularly those whose scale, timing or unpredictability threatens to overwhelm routine capabilities.^5^ Thus in MNCH, vulnerability and resilience are two divergent terms that tend to complement each other by acting as risk or protective factors, respectively, both at the individual level and at the health system. Pregnancy-related morbidity and mortality in LMICs are often preventable or treatable, but poverty, low maternal educational attainment and place of residence, among several other underlying factors, increase women’s vulnerability to adverse maternal and child health outcomes.^6–11^

Although multiple studies have examined these vulnerabilities, more attention needs to be paid to how they are patterned by gender to influence MNCH outcomes. Similarly, maternal resilience evidenced in women’s ability to sustain life satisfaction, self-esteem and purpose amidst emotional, physical and financial difficulties associated with mothering and caregiving has been studied extensively.^6 8 10–12^ However, there has been limited focus on how gender roles and norms may shape these factors.^12^

Institutionalised power, social, political and economic advantages and disadvantages afforded to different genders influence power relations. Gender also intersects with other social determinants of health, including social class, race and ethnicity,^13^ determines the hierarchy of social structure and power dynamics, and influences health outcomes. Health inequalities conditioned by gender are likely to put vulnerable populations at a further disadvantage.^14^ Today, there is an increasing need for a critical and systematic assessment of the effect of gender norms, and gender inequality on the constraints faced by and opportunities available to vulnerable populations regarding MNCH. Theoretical and conceptual advances in global health have highlighted the importance of gender expectations, roles and relations in health promotion interventions.^15–17^ For example, different gender expectations may result in greater vulnerability to mothers and children. Promising gender-sensitive practices in health have also emerged to address the HIV/AIDS epidemic and influence maternal and child health outcomes.^18–21^

The Sustainable Development Goals (SDG) aims to reduce maternal deaths to less than 70 per 100 000 live births by 2030 (SDG 3.1), neonatal mortality rate to at least as low as 12 per 1000 live births and under-5 mortality rate to at least as low as 25 per 1000 live births (SDG 3.2). These maternal and child health targets may be impossible to achieve if the critical factors shaping maternal and child health vulnerability and resilience are not well articulated. The SDG agenda must operate with gender as a cross-cutting aspect and therefore integrated within design, resource allocation, implementation, measurement and evaluation. Specifically, understanding how health systems respond to critical factors that shape the health and well-being of mothers, children and newborn is necessary.^22^

This scoping review illuminates how gender differences and relations are relevant in providing important insight into how power structures and roles aggravate vulnerability or strengthen resilience in maternal and child health in LMICs. It provides new evidence on gendered dynamics in MNCH research that must be considered as we strive to programme interventions aimed at achieving the SDG targets on maternal and child health.

## Methods

We conducted a scoping review in accordance with Arksey and O’Malley’s framework to examine the gendered dimension of vulnerability and resilience in MNCH in LMICs.^23 24^ A scoping review was necessary for a broad and comprehensive analysis without consideration of publication quality. The review followed five stages: (1) identifying the research question; (2) identifying the relevant studies; (3) selecting the studies; (4) charting data and (5) collating, summarising and reporting results.

### Identification of relevant peer-reviewed literature

This gender analysis was based on a larger scoping review aimed at developing a framework for vulnerability and resilience in MNCH in LMICs. The initial pool of literature was retrieved from major databases (ie, Medline, Embase, Scopus and Web of Science) based on a comprehensive and exhaustive search strategy that included appropriate keywords (see online supplemental appendix S1). This was supplemented by a grey literature search. The initial search was conducted on 15 January 2021 and updated on 1 March 2021.

10.1136/bmjgh-2021-007426.supp1 Supplementary data

The search strategy was structured around three blocks: (1) population (ie, MNCH, health outcomes, healthcare utilisation and social capital), (2) exposure (ie, vulnerability, resilience and high-risk) and (3) setting (ie, low-income and middle-income settings). Critical keywords and thesaurus heading terms were initially tailored to Medline and Embase searches and then adapted in other sources as necessary. Online supplemental appendix S1 shows the full search strategies for Medline and Embase.

We also reviewed reports and technical papers from multilateral and bilateral organisations, foundations, international and local non-governmental organisations, such as the Bill & Melinda Gates Foundation, Jhpiego, Clinton Health Access Initiative, International Centre for Research on Women, Women’s Health and Action Research Centre, Gender Watch and pharmacies. To gather as much evidence as possible, including high-quality literature regarding vulnerable populations in MNCH beyond the traditional sources, we incorporated the research from grey literature into this scoping review. We supplemented the database search with a bibliography search of key articles but found no relevant articles beyond what had already been extracted. We did not apply language restrictions in our search parameters and, thus, engaged translators to translate non-English publications.

### Study selection

We developed and validated a high-performance machine learning classifier/algorithm (bidirectional encoder representations from transformers) to identify relevant studies focusing on vulnerability and resilience in MNCH from an initial pool of search results. Previous studies have reported the high predictive ability of machine learning models in title and abstract screening.^25–27^ To train the machine learning algorithm, we randomly selected, screened and annotated the titles and abstracts of 500 records from the database. The performance of the model was evaluated against our classification based on precision, recall, specificity and accuracy scores. Subsequently, we applied the algorithm to review the abstracts and titles of the remaining publications to generate predictions to include or exclude them.

Covidence, an online systematic review software, was used to manage the search outputs and screening of eligible studies (https://www.covidence.org/). Two researchers screened the identified manuscripts retained from machine-learning predictions using Covidence. A third researcher reviewed and resolved all conflicts. Titles and abstracts were first screened before a full-text review for possible inclusion in the study. We included studies based on four key criteria. First, if they focus on women (pregnant/lactating and teenage mothers) and/or children (male and female) under 5 years. Second, if they focused on LMICs. We also included studies that focused on vulnerability, frailty or high risk and resilience in LMICs. Lastly, we included all study types including peer-reviewed publications, programmatic reports, and conference abstracts. There were no language restrictions nor exclusions based on the year of publication.

### Charting data

To provide a holistic gender analysis, we adapted a conceptual framework for gender analysis in health systems research by Morgan *et al*.^28^ The framework unifies several other frameworks focusing on health, health systems and development.^28^ More importantly, the framework’s unique focus on how power is constituted and negotiated makes it a valuable resource for understanding gender in terms of power relation and a source of disparity in health systems. The framework had five focal areas, namely, access to resources, division of labour, social norms, rules and decision-making, power negotiation, and structure/environment. All the articles that met the inclusion criteria for this study were further screened based on these five key gender dimensions.

Relevant data were extracted into a data collection template developed on AirTable. Articles were screened and extracted if they fit any of the five dimensions of gender and power identified in the framework. We extracted the publication metadata (ie, name of the first author, year of publication, publication title and publication country) and additional data (eg, publication type, research design and methods, study context, indices of vulnerability and resilience, and key findings from the research). Categories for the focal areas were not mutually exclusive, which means that a study could belong and be counted in more than one category where evidence of such contributions exists. During the data analysis, we grouped the articles by their specific focus on the different dimensions of gender and power relations. Table 1 presents the details of the classifications.

**Table 1:** Illustrative examples of gender analysis

What constitutes gender power relations		Illustrative gender analysis research question
Access to resources (Who has what?)	Access to resources (education, information, skills, income, employment, services, benefits, time, space, social capital, etc.)	To what extent do women and men have the same access to education, information, income, employment and other resources that contribute to improvement in maternal, newborn, and child health? Do women have sufficient means to make decisions and access healthcare services without financial restrictions?
Division of labour (Who does what?)	Division of labour within and beyond the household and everyday practices	How do women’s social roles, such as childbearing, childcare, and infant feeding, affect their economic opportunities and access to health facilities?
Social norms (How are values defined?)	Social norms, ideologies, beliefs and perceptions	How does stigma inhibit women’s access to maternal healthcare services and are these available to unmarried women and teenage mothers? How do cultural norms about motherhood put women at risk of adverse health?
Agency and decision making (Who decides?)	Agency and decision making (both formal and informal)	To what extent are women able to advocate for their health needs and contribute to household decisions that shape their and their children’s health?
Power negotiation (How is power enacted, negotiated or challenged?)	Critical consciousness, acknowledgement/lack of acknowledgement, agency/apathy, interests, historical and lived experiences, resistance or violence	How is power enacted and negotiated in relation to maternal, newborn, and child health and how does power dynamics or women’s experience of intimate partners contribute to adverse health for women, children and their families?

### Collating, synthesising and reporting the results

This review describes, first, the characteristics of the studies that meet the study inclusion criteria and, second, the findings. We report the summary statistics describing data collection methods, vulnerability/resilience context (eg, maternal or child/newborn health), gender dimension (eg, access to resources, division of labour, social norms, rules and decision making, power negotiation and structure/environment). We did not assess the quality or risk of bias for the included articles as the objective of this review was to scope and describe the breadth of gender dimensions in vulnerability or resilience in MNCH in LMICs. This review followed the Preferred Reporting Items for Systematic Reviews and Meta-Analyses extension for Scoping Reviews (PRISMA-ScR) statement guidelines to enhance transparency in reporting scoping reviews.^29^

### Patient and public involvement statement

Patients were not involved in the conduct of this study.

## Results

**Figure 1:**
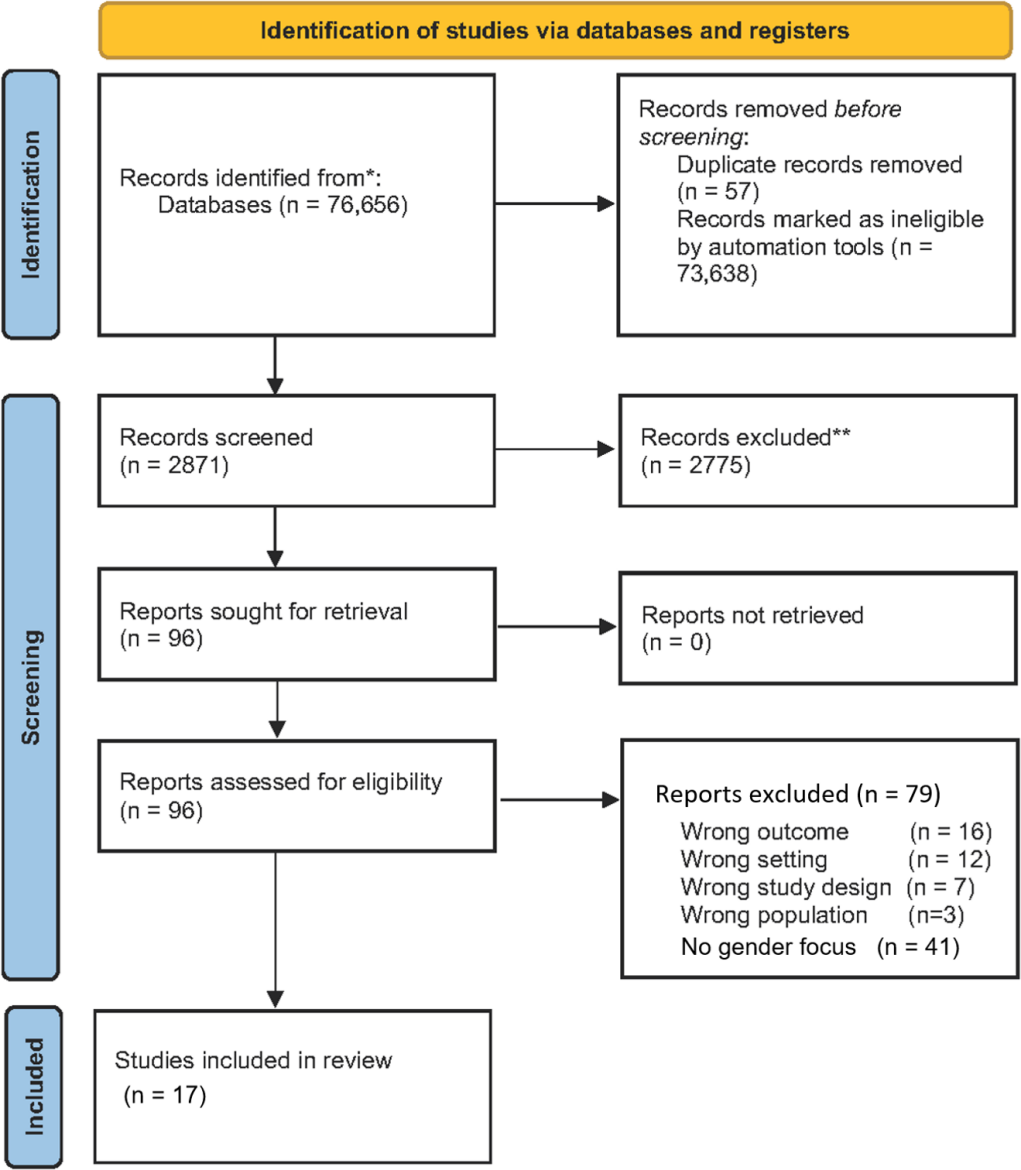
Summary of the search, selection and inclusion process.* Women (including pregnant/lactating and teenage mothers) AND/OR Children (male and female), 0-4 years OR under 5 years. (Include breastfeeding and childhood immunization studies). **Include peer-reviewed publications, programmatic reports, conference abstracts.

We identified 76 656 records through the database search (figure 1). We excluded 57 duplicate records and 73 638 abstracts that were flagged as potentially irrelevant to this study. Thereafter, we screened the remaining titles and abstracts (n=2871), we considered only 96 studies as relevant and selected them for full-text review. Of these, 79 studies did not meet the inclusion criteria and were excluded because of incorrect population, outcomes, setting, and study design or the lack of a gender focus in the analysis. Subsequently, 17 studies met our inclusion criteria for a promising gender-sensitive analysis of vulnerability and resilience in MNCH in LMICs. Online supplemental material S2 provides the details of these studies, including the year of publication, country of publication, context of the study, study design and key findings related to gender as regards vulnerability and resilience in MNCH.

10.1136/bmjgh-2021-007426.supp2 Supplementary data

### Study characteristics

**Figure 2:**
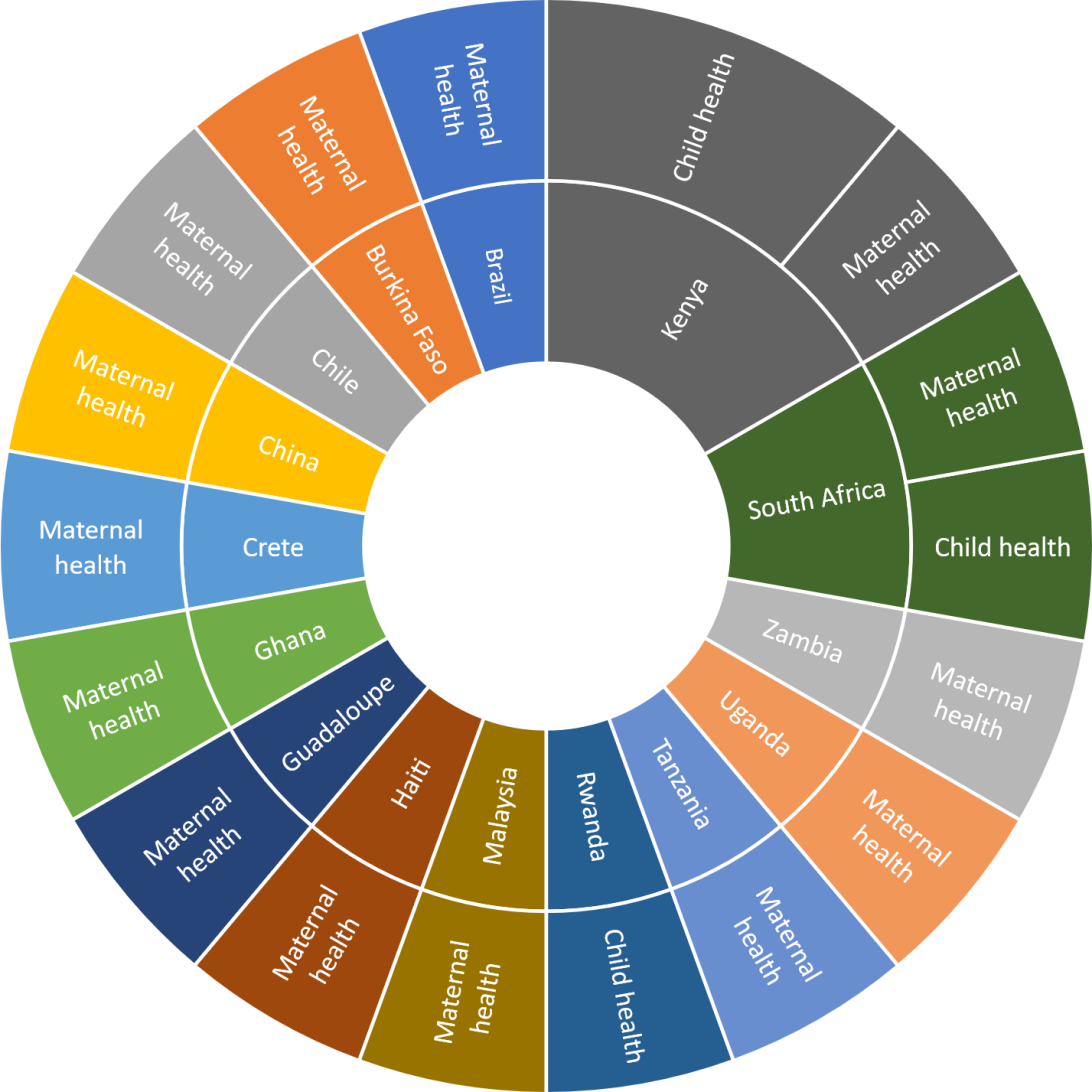
Geographical distribution of the 17 studies included in the scoping review.

A total of 17 studies met the inclusion criteria for a gender analysis. Out of these, 13 focused on maternal health and four on child health (figure 2). Eleven studies focused on sub-Saharan African countries (figure 2), of which three were from Kenya. Resilience was a more dominant focus: eight on maternal health and two on child health (online supplemental material S2).

**Figure 3:**
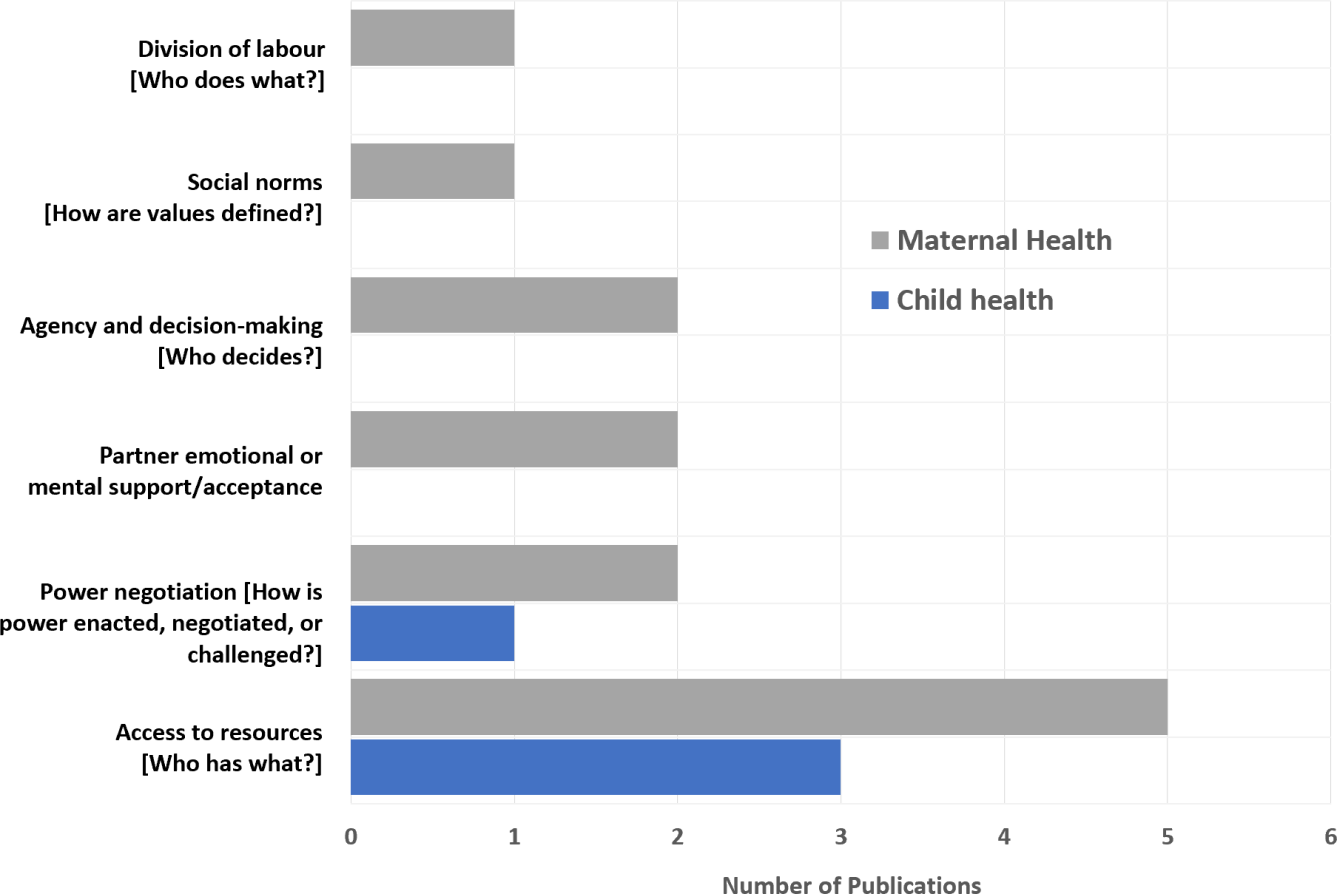
Summary of the 17 studies by gender focus and maternal or child health context.

Figure 3 presents the distribution of gender themes across maternal and child health contexts. Access to resources and decision making was the most common focus of the identified studies on both maternal (five) and child (three) health. Three studies examined power negotiation in relation to maternal (two) and child (one) health. Two studies also highlighted partner emotional or mental support in maternal health (two) and another two on the decision-making ability of mothers (two). Only a few studies examined how social norms and division of labour intersect with maternal health.

### Access to resources

Access to resources emerged as the dominant gender-focused theme (8 of 17 studies).^30–37^ In most studies, pregnant women or mothers lived in households characterised by low socioeconomic status and had lower levels of education, all of which are potentially related to poor access to maternal and child healthcare services. Among pregnant women living in a community of metropolitan Santiago, Chile,^31^ low socioeconomic status was found to be related to deteriorating reproductive, maternal and neonatal health. Warren *et al* supported this finding and found that most women affected by fistula had secondary education as the highest level of education and a very low monthly income.^36^ Most primary caretakers, including mothers, were not income earners and often relied heavily on their spouses or other household members for money.

Access to resources also emerged as an important barrier to child healthcare. For example, Johnson *et al* demonstrated that the classification as orphan and vulnerable children (OVC) directly and indirectly influenced the risk of childhood morbidity (eg, diarrhoea, fever and acute respiratory infection).^30^ This is because OVCs were more likely to be found in households headed by adults (40 years old), where the mother/caregiver had inadequate access to socioeconomic resources, such as inadequate education, and in urban areas.

Many women were often in precarious positions, relying on their spouses for financial support to access healthcare services even during emergencies. A study in Kenya reported that irrespective of marital status, having male support (eg, husband, brother or uncle), particularly financial support and help in securing transport to hospitals for care, was critical.^36^ Some women failed to attend clinics because of a lack of support from their husbands. Most husbands did not provide their wives with adequate funds for their needs during delivery.^32^ The lack of rapid access to money was another important contributing factor to a child’s deteriorating condition; it influenced the initiation of a treatment-seeking action, including where and by whom (all households) the action was performed. For instance, women in a study made many references to ‘waiting to talk to my husband’, ‘waiting to be sent money from my husband,’ and waiting for ‘his permission to pursue an action.’^37^ Such gender-reinforced inequality in access to resources could subsequently affect the health-care-seeking behaviour of mothers and, ultimately, affect childcare, especially in the context of costly maternal healthcare services.

### Division of labour

Only one study examined the dimension of the division of labour and how it intersects with maternal and child health.^38^ This study included 36 Ugandan women who were admitted with obstetric near-miss and revealed that women’s need to balance economic activities and reproduction often increased their vulnerability and ability to recover from obstetric complications. In such circumstances, social networks or social capital was generally perceived as an essential component of women’s resilience because it provides women with financial, material, and emotional assistance, including those related to household responsibilities, such as childcare.^38^

### Social norms

One study examined the dimensions of social norms in maternal health.^39^ It explored how values related to motherhood are defined and how this definition shapes or inhibits women’s access to maternal healthcare services or places women at risk of adverse health. An in-depth case study of a woman from Burkina Faso suggested that structural impediments, including motherhood and childbearing, limit individual resilience.^39^ This case study noted that the high level of social pressure on women to bear children as soon as possible, even when they are not physically or mentally capable, and the stigma associated with childlessness exacerbate maternal mortality and morbidity risks.^39^ These conditions contributed to the death of women in the case study, who could not be rescued from dying from childbirth-related complications despite having access to skilled birth attendance and emergency obstetric care.^39^

### Agency and decision making

Two studies underscored the ability of women and mothers to make informed choices and contribute to decisions related to maternal and child healthcare.^40 41^ For example, Prates *et al* showed women’s inability to adequately plan the timing of childbirth because of poor socioeconomic status and inequalities in gender power, all of which contribute to multiparity.^41^ More importantly, the existing power imbalance motivates male partner resistance to condom use as a means of family planning.^41^ Additionally, Den Hollander *et al* in Ghana underscored women’s low negotiating ability and autonomy in healthcare decision making.^40^ The study reported wide power differences between health providers and women, especially in a context shaped by authority. Women were generally uninformed about their basic health information. A high level of therapeutic misconceptions was also observed in this study. Women were also reported to rely more often on a medical professional’s opinion rather than being guided by their motivation.^40^

### Power negotiation

Power negotiation also emerged as a dominant gender dimension of vulnerability and resilience in maternal and child health. This dimension refers to how power is enacted and negotiated in relation to MNCH and how power dynamics or women’s experience of intimate partner violence contributes to adverse health for women, children and their families. Our analysis found two studies that examined power negotiation in maternal health^42 43^ and one in child health.^44^

Although seropositive status disclosure is a crucial aspect of HIV programming, women living with HIV were generally reluctant in disclosing their HIV status to their partner to avoid negative reactions from the latter, including intimate partner physical violence.^44^ Men were often not in favour of having their wives tested, fearing the indirect disclosure of their own infection.^44^ Nonetheless, partner involvement is crucial for prevention of mother-to-child transmission (PMTCT), especially because this might require mothers to use antiretroviral therapy and formula feeding for infants. The authors recommended couple counselling and partner involvement in PMTCT programmes, as only testing women can increase their susceptibility to violence despite careful counselling.

Furthermore, women’s exposure to intimate partner violence could also affect other aspects of their health. For example, Vivilaki *et al* observed that the lack of or disappointment with partner support, poor marital relationship and emotional/physical abuse had been associated with high levels of postpartum anxiety and depression.^43^ Likewise, McNaughton Reyes *et al* found that women exposed to intimate partner violence may be likely to experience persistent poor mental health across the antenatal and postnatal periods.^42^

### Partner emotional or affective support

The two studies on partner emotional or affective support were primarily related to maternal health.^45 46^ Families and partners often reacted negatively by rejecting unwed pregnant teenagers or teenage mothers.^46^ These rejections were expressed differently, including avoiding pregnant teenagers or verbal abuse.^46^ The analysis suggested that low-resilient women with threatened premature labour reported higher pressures from child support concerns after delivery, less active coping, less positive affect and more negative affect.^45^

## Discussion

This scoping review illuminates the gendered dynamics of vulnerability and resilience in MNCH research. Based on the 17 studies reviewed, we found that gender norms, roles and relationships significantly influence and reinforce vulnerability and resilience in maternal and child health. The role of gender-transformative interventions cannot be overemphasised in addressing these societal structures and widely held social values that perpetuate the gender inequities identified in this review. Our work highlights some promising gender-transformative interventions that should be prioritised in addressing vulnerabilities in MNCH (see table 2 for the summary). These are potential interventions based on the problems identified. Most importantly, women should have unhindered access to maternal and child healthcare services regardless of education, level of wealth, age or marriage.

**Table 2:** Implications and recommendations for programme and policy

Gender aspect	Recommendations
Access to resources (Who has what?)	Eliminate financial barriers in accessing maternal and child health services. Empower mothers through formal and informal education to enhance their health awareness and consciousnesss, efficacy and ability to make informed decisions about their health and their child/children’s health.
Division of labour (Who does what?)	Provide adequate support and affordable childcare for mothers to enhance their productivity and participation in the labour force. Incentivise programmes that motivate the involvement of men in childcare and house chores.
Social norms (How are values defined?)	Address issues regarding cultural stereotypes that impede maternal access to healthcare services, including those related to marriage and adolescent motherhood. This could be in the form of providing a friendly and safe environment for adolescent and unmarried mothers to access healthcare. Engage community leaders in alleviating social norms that put women and girls at risk of poor health. This includes social norms that limit the contributions of women beyond motherhood.
Agency and decision making (Who decides?)	Provide universal access to safe and effective means of contraception, irrespective of the level of education and wealth. Strengthen the capacity of women and girls through education and job creation to contribute significantly to household decision making. Empower women to make decisive decisions about whether they want to have a/another baby and when they want to do so.
Power negotiation (How is power enacted, negotiated or challenged?)	Develop effective systems and strategies for the reporting and management of intimate partner violence and abuse.

As highlighted in this review, access to resources was a dominant theme in 8 of the 17 reviewed studies.^30–37^ Mothers in most of the studies reported having to wait for their husbands or other relatives for funds before they could access healthcare services. This process could pose a significant threat to them and their children’s health and well-being, especially during emergencies. Women’s access to healthcare services is compounded by socio-cultural stereotypes that impede maternal access to healthcare services, including marriage and adolescent motherhood. Multiple studies have highlighted how cultural stereotypes and stigma may hinder healthcare access for the same people who need the service the most.^47^ In some cultural settings, unmarried women and adolescent mothers are unable to access care, partly because of the emphasis on marriage and motherhood in many African societies. Many women in search of assistance have fallen victims of human trafficking rings in baby factories where their babies are sold, and then they have been held against their will, thereby compounding their woes.^48 49^

However, these barriers to healthcare access can be alleviated through a multisectoral intervention that addresses sociocultural stereotypes and the high costs of access to health services, including the cost of registration, treatment and care. For example, in Nigeria, the removal of user fees and increased community engagement for the most vulnerable is associated with a higher level of maternal health-seeking behaviour.^50^ Similar findings have been reported in other LMICs, including China, Zambia, Jamaica and India.^51 52^

Although the abolition of user fee policies is necessary to achieve universal access to quality healthcare, multiple studies have underscored that such policies are not sufficient to improve maternal healthcare utilisation.^53 54^ The removal of user fees may increase uptake but may not reduce mortality proportionally if the quality of facility-based care is poor.^55^ This may especially be salient in settings where healthcare access is limited by structural barriers related to the distance of health facilities or cost of transportation, waiting times and other additional costs.^56 57^ Masiye *et al* emphasised that the cost of transportation is mainly responsible for limiting the protective effect of user fee removal on catastrophic healthcare among the poorest households.^57^ This finding is supported by Dahab and Sakellariou, who identified transportation barriers as among the most important barriers to maternal health in low-income African countries.^56^ In fact, one study in our review reported that receiving financial support and helping in securing transport to hospitals for healthcare is critical.^36^

Previous studies have also highlighted that poorly implemented user fee removal policies benefit more well-off women than poor ones, and in cases where there are significant immediate effects on the uptake of facility delivery, this trend is not sustained over time.^58 59^ Given these findings, there is an overarching need for comprehensive and multisectoral approaches to achieve sustainable improvements in maternal health. In some studies, women who received financial incentives as a part of neonatal care or conditional cash transfers reported better healthcare-seeking behaviours than those who did not.^60^ Morgan *et al* emphasised that financial incentives can increase the quantity and quality of maternal health services and address health systems and financial barriers that prevent women from accessing and providers from delivering quality and lifesaving maternal healthcare.^60^ There is also an increasing consensus on the need to engage the community and religious leaders in challenging many of the cultural impediments to healthcare access. Countries in which these have been attempted have reported huge successes in improving healthcare access and service utilisation.

In several LMICs, women are tasked with the responsibility of childbearing and child-rearing; both could significantly affect women’s economic productivity. Empowering women through skill acquisition could also offer a viable financial alternative and alleviate the high cost of accessing healthcare services, especially for women in low socioeconomic strata. Adequate incentives and support for mothers of children could also significantly ease the pressure on women to balance motherhood and economic activities. Some studies have reported the positive effects of programmes that help women with childcare.^61 62^ Such empowerment programmes could also be extended to single women and women in sole-based or female-headed households, because these family types are characterised by low levels of education and household wealth.

Another important gender dimension is the need for women and mothers to make decisions about their health and well-being. As highlighted in our review, women have limited contribution to decision-making processes that are related to healthcare and family planning.^40 41^ This limitation is complicated by power imbalances between women and their spouses and between women and healthcare workers.^40 41^ One study found that women are only aware of condoms as a means of contraception and that their male partners resist to use condom. However, they are unwilling to use other means of contraception, perhaps because of the known or perceived side effects. Family planning services must be integrated into existing maternal and child health programmes, so that women are adequately equipped with sexual and reproductive health information and have the autonomy to choose their preferred means of contraception with minimal effects on pleasure.

Male partner involvement is also crucial for PMTCT of HIV, especially because this requires mothers to use antiretroviral therapy and feed the child using formula feeding.^44^ Although the involvement of the spouse during childbirth and child-rearing could alleviate some of the economic implications of motherhood, unfortunately, many male partners are not usually involved in childcare.^63^ A few studies in our review reported on women’s experiences of intimate partner violence and how this intersects with maternal and child vulnerabilities.^42–44^ Women’s exposure to intimate partner violence is associated with high levels of postpartum anxiety and depression and their experience of persistent poor mental health across the antenatal and postnatal periods.^42 43^ The fear of intimate partner violence has also been reported to influence women’s disclosure of HIV status to their spouses.^44^ This occurs especially because men are often not favouring having their wives tested, fearing the indirect disclosure of their own infection. As recommended by Gaillard *et al*^44^ and other scholars,^64^ the continued counselling of women alone may not eliminate some of the maternal risks of intimate partner violence. However, MNCH programmes could alleviate these risks through couple-counselling and partner involvement in PMTCT programmes.

Aside from increasing male partner involvement in reducing maternal risks of intimate partner violence, the development of effective systems and strategies for the reporting and management of intimate partner violence and abuse is important. Many LMICs have legal structures for seeking redress for intimate partner violence; however, reporting the same has not been effective. Multiple studies have examined women’s motivation to remain in violent unions.^65–68^ The findings of these studies, among several others, have highlighted the subsistence and stereotypes associated with being divorced, among others. As a result, strong systems may especially be important for women in low socioeconomic status who must remain in violent marriages for survival. Altogether, these findings have pointed to the need for context and a women-centric perspective in developing strategies to eliminate violence against women, as such strategies may be inefficient if they do not address some of the bottlenecks for combating violence against women. Some studies have reported the effectiveness of women’s social empowerment combined with economic empowerment in reducing women’s vulnerabilities to intimate partner violence.^69^ Such interventions may also provide women with resources to access healthcare services and alleviate maternal experiences of intimate partner violence. However, these interventions could aggravate experiences of intimate partner violence, especially in settings where maternal empowerment is perceived to threaten established gender norms.^70–72^ Nonetheless, multiple studies in Tanzania have reported that maternal empowerment has led to considerable reductions in physical intimate partner violence and posed no additional adverse health risks.^69^

Watts and Mayhew^73^ and García-Moreno *et al*^74^ recommended a more active approach, that is, to integrate health systems response into maternal and child healthcare. Today, there is a global consensus to strengthen healthcare professionals’ ability to identify victims of intimate partner violence and provide first-line supportive care and referral to other care services.^74^ A functional and well-financed health system is also important to prevent violence against women and respond to victims and survivors in a consistent, safe, and effective manner to enhance their health and well-being.^74^ Health providers could probe women about their experiences of violence or evaluate them for any potential indicator of partner violence, such as any history of unexplained injury or maternal bleeding, preterm labour or birth, and foetal injury or death.^73^ The healthcare system can also provide women with a safe environment in which they can confidentially disclose experiences of violence and receive a supportive response.

Although our review addresses an important gap in the literature, it is not without limitation. The first is that the inclusion of articles in this review is based solely on their focus on vulnerability or resilience in LMICS. Therefore, studies on vulnerability or resilience outside of LMICs, in locations where pockets of vulnerable populations occur in high-income nations have not been captured. Additionally, while we made every attempt to find all accessible material, it is possible that we omitted some publications with distinct perspectives that were not represented in the review’s evidence from grey literature particularly, given how broad it is.

## Conclusion

Only a few studies have examined vulnerability and resilience in maternal and child health, especially in LMICs. We have identified some gendered dynamics of vulnerability and resilience in MNCH through this scoping review. Findings from this scoping review suggest that there is a great need to continue to empower women and mothers to access resources, contribute to decisions about their own health, and eliminate structural or social stereotypes that limit their agency.

## Data Availability

All data relevant to the study are included in the article or uploaded as online supplemental information.
