# Supplementary material for: Examining vulnerability and resilience in maternal, newborn and child health through a gender lens in low-income and middle-income countries: a scoping review
Source: BMJ Glob Health. 2022 Apr 19;7(4):e007426. doi: 10.1136/bmjgh-2021-007426 (PMC9024279; doi:10.1136/bmjgh-2021-007426)
Supplement: Supplementary data [file bmjgh-2021-007426supp001.pdf]

Appendix 1: Database search strategies

Maternal Health

'maternal health'.ti,ab,kw.
('pregnancy'.ti,ab,kw. OR 'adolescent pregnancy'.ti,ab,kw. OR 'first trimester pregnancy'.ti,ab,kw. OR 'multiple pregnancy'.ti,ab,kw. OR 'twin pregnancy'.ti,ab,kw. OR 'second trimester pregnancy'.ti,ab,kw. OR 'third trimester pregnancy'.ti,ab,kw. OR 'unplanned pregnancy'.ti,ab,kw. OR 'unwanted pregnancy'.ti,ab,kw.
((pregnan*) adj3 (adolescen* OR teen* OR multip* OR twin* OR quad* OR quin* OR trip* OR trimester OR early OR late OR unplanned OR unintended OR unwanted OR undesired OR intrauterine OR maintenance))
(twinning* OR 'multiple birth’ OR quadruplet* OR quintuplet* OR triplet* OR superfetation OR 'child bearing’ OR childbearing OR gestation OR gravidity OR 'labor presentation’ OR ((trimester) adj3 (first OR second OR third OR 1st OR 2nd OR 3rd OR mid*)).ti,ab,kw.
Pregnancy in Adolescence/
(pregnan* adj2 (adolescen* or teen* or schoolchild*)).ti,ab,kw.
Pregnancy, unplanned/ or Pregnancy, unwanted/
pregnan* adj3 (prevent* or interrupt* or unplanned or unwanted or mistimed)).ti,ab,kw.
Prenatal Care/
(((antenatal or ante-natal or prenatal or pre-natal or antepartum or ante-partum) adj3 (care or service* or counsel* or test*)) or (birth adj3 prepar*)).ti,ab,kw.
Maternal Health Services/
((maternal or mother*) adj3 (health or service* or care)).ti,ab,kw.
Midwifery/
(midwi* or skilled birth or skilled attendan*).ti,ab,kw.
Postnatal Care/
Perinatal Care/
Postpartum Period/
((postnatal or post-natal or perinatal or peri-natal or postpartum or post-partum) adj2 (care or service*)).ti,ab,kw.
Maternal Nutritional Physiological Phenomena/
Prenatal Nutritional Physiological Phenomena/
Breast Feeding/
(breast feed* or breast fed or breastfeed* or breastfed).ti,ab,kw.

Sule FA, *et al. BMJ Global Health* 2022; 7:e007426. doi: 10.1136/bmjgh-2021-007426

Child health

(Infant health)) OR (Infant Services, Health)) OR (Service, Child Health)) OR (Infant Health Service)) OR (Neonate)) OR (Neonate)) OR (Neonates)) OR (Newborn Infants)) OR (Infants, Newborn) (Infant, Premature/ or Infant, Newborn/ or Infant, Low Birth Weight/ or Infant, Extremely Low Birth Weight/ or Infant, Small for Gestational Age/ or Infant/ or Infant, Very Low Birth Weight/ or Infant, Postmature/ or Infant, Extremely Premature/) and Early Diagnosis/
(early adj1 diagnos* adj2 (infant* or neonat* or newborn*)).ti,ab,kw.
diagnosis.fs. and (infant* or neonat* or newborn*).ti,ab,kw.
(Immunization/ or Immunization, passive/ or Immunization schedule/ or Immunization, secondary/ or Immunization Programs/ or Vaccination/ or Mass vaccination/) and (Infant, Premature/ or Infant, Newborn/ or Infant, Low Birth Weight/ or Infant, Extremely Low Birth Weight/ or Infant, Small for Gestational Age/ or Infant/ or Infant, Very Low Birth Weight/ or Infant, Postmature/ or Infant, Extremely Premature/ or Child/ or Child, Preschool/ or Adolescent/ or Pregnancy/)
((immuniz* or immunis* or vaccinat*) and (infant* or newborn* or neonat* or child* or adolescen* or teen*)).ti,ab,kw.
Child health services/ or Maternal-child health services/
exp child nutrition disorders/ or exp infant nutrition disorders/ or (exp nutrition disorders/ and (exp Infant/ or Child, Preschool/))
'Delivery of Health Care, Integrated'/
((integrat* adj3 (health care or healthcare or management or treat* or service*) adj3 (child* or schoolchild* or infant* or neonat* or newborn or adolescen* or teen*)) or IMCI or IMNCI).ti,ab,kw.
(Guideline Adherence/ or Quality Assurance, Health Care/) and (exp Infant/ or Child, Preschool/)
((((guideline* or protocol*) adj3 (adher* or observ*)) or "prescribed care") and (infant* or newborn* or neonat* or child*)).ti,ab,kw.
(Diarrhea/di, dt, ep, pc, th, tm or Diarrhea, Infantile/di, dt, ep, pc, th, tm) and (Infant, Premature/ or Infant, Newborn/ or Infant, Low Birth Weight/ or Infant, Extremely Low Birth Weight/ or Infant, Small for Gestational Age/ or Infant/ or Infant, Very Low Birth Weight/ or Infant, Postmature/ or Infant, Extremely Premature/ or Child/ or Child, Preschool/ or Adolescent/)
(diarrh* and (infant* or newborn* or neonat* or child* or schoolchild* or adolescen* or teen*)).ti,ab,kw.
Fluid Therapy/ and (Infant, Premature/ or Infant, Newborn/ or Infant, Low Birth Weight/ or Infant, Extremely Low Birth Weight/ or Infant, Small for Gestational Age/ or Infant/ or Infant, Very Low Birth Weight/ or Infant, Postmature/ or Infant, Extremely Premature/ or Child/ or Child, Preschool/)
Child Development/ or Adolescent Development/

Health utilisation

health care system'.ti,ab,kw.
'health care access'.ti,ab,kw.
'health care availability'.ti,ab,kw.
'health care disparity'.ti,ab,kw.
'health care distribution'.ti,ab,kw.
'health care need'.ti,ab,kw.
'health care planning'.ti,ab,kw.
'healthcare system'.ti,ab,kw.
'health disparity'.ti,ab,kw.
'health care delivery'.ti,ab,kw.
'ambulatory care'.ti,ab,kw.
'hospital care'.ti,ab,kw.
'primary health care'.ti,ab,kw.
'secondary health care'.ti,ab,kw.
'tertiary care center'.ti,ab,kw.
'health care quality'.ti,ab,kw.
((health* OR service*) adj3 (access* OR availab* OR disparat* OR equit* OR inequal* OR plan* OR priorit* OR resource* OR suppl* OR deliver*)).ti,ab,kw.

Health Outcomes

Obstetric Labor Complications/
Pregnancy Complications/
stillbirth.ti,ab,kw.
stillborn.ti,ab,kw.
((obstetric* or pregnan* or labour or labor or parturition) adj3 (emergenc* or complication*)).ti,ab,kw.
('maternal morbidity'.ti,ab,kw. OR 'maternal mortality'.ti,ab,kw. OR 'perinatal death'.ti,ab,kw. OR 'perinatal morbidity'/exp OR 'newborn morbidity'.ti,ab,kw. OR 'newborn death'.ti,ab,kw. OR 'child death'.ti,ab,kw. OR 'infant mortality'.ti,ab,kw. OR 'small for date infant'.ti,ab,kw. OR 'low birth weight'.ti,ab,kw. OR 'extremely low birth weight'.ti,ab,kw. OR 'very low birth weight'.ti,ab,kw. OR 'apgar score'.ti,ab,kw.
((maternal OR mother OR perinatal OR neonat* OR infant*) NEAR/3 (death OR mortality OR morbidity OR disease)) OR 'small for gestational age' OR ((SGA OR 'small for gestational age' OR 'small for date' OR 'small for age') adj3 (infant* OR neonat* OR newborn OR baby))
(('ELBW' OR 'VLBW' OR 'LBW' OR (('birth weight' OR birthweight OR 'LBW' OR underweight) adj3 (infant* OR neonat* OR newborn OR low))
((apgar OR APGAR) adj3 (classif* OR coeff* OR index* OR rating OR scale OR test OR timer OR score)).ti,ab,kw.
((child* or schoolchild* or adolescen* or teen*) adj2 (develop* or progress*)).ti,ab,kw.
Pregnancy in Adolescence/
Kangaroo-Mother Care Method/
(kangaroo adj2 (mother or infant or care)).ti,ab,kw.
(Anemia/dt, pc or Anemia, Hypochromic/dt, pc or Anemia, Iron-Deficiency/dt, pc) and Pregnancy/
((maternal or mother* or pregnan*) adj2 (nutrition* or folate or folic or iron or anaemi* or anemi*)).ti,ab,kw.
(Malaria/di, dt, pc or Malaria, Falciparum/di, dt, pc or Malaria, Vivax/di, dt, pc) and (Pregnancy/ or Pregnancy Complications, Parasitic/)
((malaria* or falciparum or vivax) adj3 (pregnan* or mother* or maternal or postpartum or postpartum)).ti,ab,kw.
Mental health/ or Mental disorders/ or Mental health services/ or Community mental health services/
Maternal behavior/ or Mother-child relations/ or Parenting/ or Paternal behavior/
Depression, Postpartum/
(((mental or behavio*) adj3 (health or disorder*)) or postpartum depression or post-partum depression).ti,ab,kw.

Social capital

'social determinants of health'.ti,ab,kw.<br>'neighborhood'.ti,ab,kw. OR 'community'.ti,ab,kw. OR 'religion'.ti,ab,kw. OR 'occupation'.ti,ab,kw.<br>'employment'/exp OR 'income'.ti,ab,kw. OR 'ethnicity'.ti,ab,kw. OR 'race'.ti,ab,kw. OR 'education'.ti,ab,kw.<br>'social status'/exp OR 'lowest income group'.ti,ab,kw. OR 'poverty'.ti,ab,kw. OR 'social background'.ti,ab,kw.<br>'social class'.ti,ab,kw. OR 'social network'.ti,ab,kw. OR 'social capital'.ti,ab,kw. OR 'health literacy'.ti,ab,kw. OR 'locus of control'.ti,ab,kw.<br>'attitude to health'.ti,ab,kw. OR 'health insurance'.ti,ab,kw. OR 'private insurance'.ti,ab,kw. OR 'public health insurance'.ti,ab,kw. OR<br>'social stigma'.ti,ab,kw. OR 'stigma'.ti,ab,kw.<br>'risk perception' OR 'medically uninsured'.ti,ab,kw. OR 'risk factor'.ti,ab,kw. OR 'relative risk'<br>((health*) adj3 (knowledge OR attitude* OR practice OR accept* OR barrier))<br>'HLC' OR 'health locus of control' OR uninsured OR 'insurance status'<br>neighborhood OR neighbourhood OR 'poverty areas' OR 'religious belief' OR spiritual* OR profession OR 'employment status'<br>income OR 'social network' OR 'sociocultural class' OR 'socioeconomic class' OR residenc*<br>(education*) adj3 (achievement OR attain* OR level))<br>((social*) adj3 (determin* OR factor))<br>((underserve*) adj3 (neighborhood OR neighbourhood OR area))<br>((social* OR socio* OR religious OR gender OR politic* OR econom*) NEAR/3 (barrier OR threshold)).ti,ab,kw.)

Resource limited setting

'developing countries'.ti. OR 'low-income countries'.ti. OR 'middle-income country'.ti. OR lmic.ti. OR lmics.ti. OR 'less-developed countries'.ti. OR 'under-developed nations'.ti. OR 'third-world country'.ti. OR 'third-world nations'.ti.

(afghanistan OR albania OR algeria OR american samoa OR angola OR "antigua and barbuda"
OR antigua OR barbuda OR argentina OR armenia OR armenian OR aruba OR azerbaijan OR bahrain OR bangladesh OR barbados OR republic
of belarus OR belarus OR byelarus OR belorussia OR byelorussian OR belize OR british honduras OR benin OR dahomey OR bhutan OR bolivia OR "bosnia and herzegovina"
OR bosnia OR herzegovina OR botswana OR bechuanaland OR brazil OR brasil OR bulgaria OR burkina faso OR burkina fasso OR upper volta OR burundi OR urundi OR cabo verde OR
cape verde OR cambodia OR kampuchea OR khmer republic OR cameroon OR cameron OR cameroun OR central african republic OR ubangi shari OR chad OR chile OR china OR colombia OR comoros OR comoro islands
OR iles comores OR mayotte OR democratic republic of the congo OR democratic republic congo OR congo OR zaire OR costa rica OR "cote d’ivoire" OR "cote d’ ivoire" OR cote divoire OR cote d ivoire OR ivory coast
OR croatia OR cuba OR cyprus OR czech republic OR czechoslovakia OR djibouti OR french somaliland OR dominica OR dominican republic OR ecuador OR egypt OR united arab republic OR el salvador OR equatorial guinea
OR spanish guinea OR eritrea OR estonia OR eswatini OR swaziland OR ethiopia OR fiji OR gabon OR gabonese republic OR gambia OR "georgia (republic)" OR georgian OR ghana OR gold coast
OR gibraltar OR greece OR grenada OR guam OR guatemala OR guinea OR guinea bissau OR guyana OR british guiana OR haiti OR hispaniola OR honduras OR hungary OR india OR indonesia OR timor OR iran OR iraq OR isle of man OR jamaica OR jordan OR kazakhstan OR kazakh OR kenya OR "democratic people’s republic of korea" OR republic of korea OR north korea OR
south korea OR korea OR kosovo OR kyrgyzstan OR kirghizia OR kirgizstan OR kyrgyz republic OR kirghiz OR laos OR lao pdr OR "lao people's democratic republic" OR latvia OR lebanon OR lebanese republic
OR lesotho OR basutoland OR liberia OR libya OR libyan arab jamahiriya OR lithuania OR macau OR macao OR republic of north macedonia OR macedonia OR madagascar OR malagasy republic
OR malawi OR nyasaland OR malaysia OR malay federation OR malaya federation OR maldives OR indian ocean islands OR indian ocean OR mali OR malta OR micronesia OR federated states
of micronesia OR kiribati OR marshall islands OR nauru OR northern mariana islands
OR palau OR tuvalu OR mauritania OR mauritius OR mexico OR moldova OR moldovian OR mongolia OR montenegro OR morocco OR ifni OR mozambique OR portuguese east africa OR myanmar OR burma OR namibia OR nep al OR netherlands antilles OR nicaragua OR niger OR nigeria OR oman OR muscat OR pakistan OR panama OR papua new guinea OR new guinea
OR paraguay OR peru OR philippines OR philipines OR phillipines OR phillippines OR poland OR "polish people's republic" OR portugal OR portuguese republic OR puerto rico OR romania OR russia OR russian federation
OR ussr OR soviet union OR union of soviet socialist republics OR rwanda OR ruanda OR samoa OR pacific islands OR polynesia OR samoan islands OR navigator island OR navigator islands OR "sao tome and principe"
OR saudi arabia OR senegal OR serbia OR seychelles OR sierra leone OR slovakia OR slovak republic OR slovenia OR melanesia OR solomon island OR solomon islands OR norfolk island OR norfolk islands OR somalia OR
south africa OR south sudan OR sri lanka OR ceylon OR "saint kitts and nevis" OR "st. kitts and nevis" OR saint lucia OR "st. lucia" OR "saint vincent and the grenadines" OR saint vincent OR "st. vincent" OR grenadines
OR sudan OR suriname OR surinam OR dutch guiana OR netherlands guiana OR syria OR syrian arab republic
OR tajikistan OR tadjikistan OR tadzhikistan OR tadzhik OR tanzania OR tanganyika OR thailand OR siam OR timor leste OR east timor OR togo OR togolese republic OR tonga OR "trinidad and tobago"
OR trinidad OR tobago OR tunisia OR turkey OR turkmenistan OR turkmen OR uganda OR ukraine OR uruguay OR uzbekistan OR uzbek OR vanuatu OR new hebrides OR venezuela OR vietnam OR viet nam OR middle east OR west bank OR gaza OR palestine OR yemen OR yugoslavia OR zambia OR zimbabwe OR northern rhodesia OR global south OR africa south of the sahara OR sub-saharan africa OR subsaharan africa OR africa, central OR central africa OR africa, northern OR north africa OR northern africa OR magreb OR maghrib OR sahara OR africa, southern OR southern africa OR africa, eastern OR east africa OR eastern africa OR africa, western OR
west africa OR western africa OR west indies OR indian ocean islands OR caribbean OR central america OR latin america OR "south and central america" OR south america OR asia, central OR central asia OR asia, northern OR north asia OR northern asia OR asia, southeastern OR southeastern asia OR south eastern asia OR southeast asia OR south east asia OR asia, western OR western asia OR europe, eastern OR east europe OR eastern europe OR developing country OR developing countries OR developing nation? OR developing population? OR developing world OR less developed countr* OR less developed nation? OR less developed population? OR less developed world OR lesser developed countr* OR lesser developed nation? OR lesser developed population? OR lesser developed world OR under developed countr* OR under developed nation? OR under developed population?
OR under developed world OR underdeveloped countr* OR underdeveloped nation? OR underdeveloped population? OR underdeveloped world OR middle income countr* OR middle income nation? OR middle
income population? OR low income countr* OR low income nation? OR low income population? OR lower income countr* OR lower income nation? OR lower income population? OR underserved countr* OR underserved nation? OR underserved population? OR underserved world OR under served countr* OR under served nation? OR under served population? OR under served world OR deprived countr* OR deprived nation? OR deprived population? OR deprived world OR poor countr* OR poor nation? OR poor population? OR poor world OR poorer countr* OR poorer nation? OR poorer population? OR poorer world OR developing econom* OR less developed econom* OR lesser developed econom* OR under developed econom* OR underdeveloped econom* OR middle income econom* OR low income econom* OR lower income econom* OR low gdp OR low gnp OR low gross domestic OR low gross national OR lower gdp OR lower gnp OR lower gross domestic OR lower gross national OR lmic OR lmics OR third world OR lami countr* OR transitional countr* OR emerging economies OR emerging nation?).ti,ab,sh,kf.

(afghan OR afghans OR afghani OR albanian? algerian? OR american samoan? OR angolan? OR antiguan? OR barbudan? OR argentine? OR argentinian? OR argentinean? OR armenian? OR aruban? OR azerbaijani?
OR bahraini? OR bangladeshi? OR bangalees OR bajan? OR belarusian? OR byelorussian? OR belizean? OR beninese? OR bhutanese OR bolivian? OR bosnian? OR botswana OR batswana OR brazilian? OR brasilian?
OR bulgarian? OR burkinabe OR burkinese OR burundian? OR cape verdean? OR cabo verdean? OR cambodian? OR khmer OR cameroonian? OR central african? OR chadian? OR chilean? OR chinese OR colombian?
OR comorian? OR congolese OR costa rican? OR ivorian? OR croatian? OR cuban? OR cypriot? OR czech? OR djiboutian? OR dominican? OR ecuadorian? OR egyptian? OR salvadoran? OR equatorial guinean?
OR equatoguinean? OR eritrean? OR estonian? OR swazi? OR swati? OR ethiopian? OR fijian OR gabonese OR gabonaise OR gambian? OR georgian? OR ghanaian? OR gibraltarian? OR greek? OR grenadian? OR guamanian?
OR guatemalan? OR guinean? OR bissau guinean? OR guyanese OR haitian? OR honduran? OR hungarian? OR indian? OR indonesian? OR iranian? OR iraqian? OR iraqi? OR manx OR jamaican? OR jordanian? OR kazakhstani?
OR kenyan? OR kirabati OR kirabatian? OR north korean? OR korean? OR kosovar? OR kosovan? OR kyrgyz* OR lao OR laotian? OR latvian? OR lebanese OR lesothan? OR lesothonian? OR mosotho OR basotho OR liberian?
OR libyan? OR lithuanian? OR macanese OR macedonian? OR malagasy OR madagascan? OR malawian? OR malaysian? OR maldivian? OR malian? OR maltese OR marshallese? OR mauritanian? OR mauritian? OR mexican?
OR micronesian? OR moldovan? OR mongolian? OR mongol OR montenegrin? OR moroccan? OR mozambican? OR burmese OR myanma OR namibian? OR nauruan? OR nepali OR nepalese OR netherlands antillean?
OR nicaraguan? OR nigerien? OR nigerian? OR northern mariana islander? OR mariana? OR omani? OR pakistani? OR palauan? OR panamanian? OR papua new guinean? OR paraguayan? OR peruvian? OR philippine?
OR philipine? OR phillipine? OR phillippine? OR filipino? OR filipina? OR polish OR pole OR poles OR portuguese OR puerto rican? OR romanian? OR russian? OR soviet people OR soviet population OR rwandan?
OR rwandese OR ruandan? OR ruandese OR samoan? OR sao tomean? OR santomean? OR saudi arabian? OR saudi? OR senegalese OR serbian? OR montenegrin? OR seychellois OR seychelloise? OR sierra leonean?
