# Supplementary material for: Examining vulnerability and resilience in maternal, newborn and child health through a gender lens in low-income and middle-income countries: a scoping review
Source: BMJ Glob Health. 2022 Apr 19;7(4):e007426. doi: 10.1136/bmjgh-2021-007426 (PMC9024279; doi:10.1136/bmjgh-2021-007426)
Supplement: Supplementary data [file bmjgh-2021-007426supp002.pdf]

Supplementary file 2: Full details of the 17 studies included in the scoping review

S/N	Author Year	Publication Title	Country [Context]	Main-Theme	Main Findings
1	Rotheram-Borus et al. (2009)	Maternal HIV does not affect resiliency among uninfected/HIV exposed South African children from birth to 5 years of age	South Africa [Child health]	Access to resources [Who has what?]	Resiliency was significantly associated with lower income, food security, not having a live-in partner, and the absence of maternal risk (i.e. not being depressed, using alcohol, or being a victim of intimate partner violence).
2	Jana et al. (2013)	Mental health predicted by coping, social support, and resilience among young unwed pregnant Malaysian women and mothers living in shelter homes	Malaysia [Maternal health]	Partner emotional or mental support/acceptance	Families and partners often reacted to the pregnancy by rejection of the unwed pregnant teenagers or teenage mothers. These rejections took different expressions in terms of avoiding them or verbal harassment.
3	Vivilaki et al. (2016)	Maternal screening for early postnatal vulnerability	Crete [Maternal health]	Power negotiation [How is power enacted, negotiated, or challenged?]	The lack of or disappointment with partner support, poor marital relationship, and emotional/physical abuse have been associated with high levels of postpartum anxiety and depression.
4	Nie et al. (2017)	The impact of resilience on psychological outcomes in women with threatened premature labor and spouses: a cross-sectional study in Southwest China	China [Maternal health]	Partner emotional or mental support/acceptance	The analysis suggested that low resilient women with threatened premature labour reported higher pressures from concerns of child support after delivery, less active coping, less positive affect, and more negative affect. High resilient women reported more social support.
5	Scheidell et al. (2018)	Socioeconomic vulnerability and sexually transmitted infection among pregnant Haitian women	Haiti [Maternal health]	Access to resources [Who has what?]	The study findings highlighted how socioeconomic vulnerabilities, such as low levels of women’s education, increase their risk of early sexual debut.

S/N	Author Year	Publication Title	Country [Context]	Main-Theme	Main Findings
6	Kaye et al. (2014)	Survivors' understanding of vulnerability and resilience to maternal near-miss obstetric events in Uganda	Uganda [Maternal health]	Division of labour [Who does what?]	The study highlighted how women's need to balance economic activities and reproduction increased their vulnerability and ability to recover from obstetric complications. In such circumstances, social networks or social capital was generally perceived as an essential component of women's resilience because it provided women with the financial, material, and emotional assistance, including those related to household responsibilities, such as childcare.
7	Johnson et al. (2010)	Orphanhood and vulnerability: a conduit to poor child health outcomes in Rwanda	Rwanda [Child health]	Access to resources [Who has what?]	The study showed that the orphan and vulnerable children (OVC) status directly and indirectly influenced the risk of childhood morbidity (e.g., diarrhoea, fever, and acute respiratory infection [ARI] symptoms). This is because OVCs were more likely to be found in households headed by older adults (40 years old) and females, where the mother/carer has inadequate access to socioeconomic infrastructures (e.g. inadequate education), and that are more likely to be in urban areas.
8	Gaillard et al. (2002)	Vulnerability of women in an African setting: lessons for mother-to-child HIV transmission prevention programmes	Kenya [Child health]	Power negotiation [How is power enacted, negotiated, or challenged?]	Women were generally reluctant about disclosing their HIV status to their partner. Violence against women living with HIV was reported in this study. This was primarily linked to women's disclosure of their seropositivity status. Men were often not in favour of having their wives tested, fearing the indirect disclosure of their own infection. The authors recommended couple-counselling and partner involvement in mother-to-child-transmission (MTCT) prevention programmes, as only testing women can increase their susceptibility to violence despite careful counselling. Partner support is crucial for preventing MTCT, especially because this requires mothers to use antiretroviral therapy and feed the child using formula feeding.

S/N	Author Year	Publication Title	Country [Context]	Main-Theme	Main Findings
9	Zakayo et al. (2020)	Vulnerability and agency across treatment-seeking journeys for acutely ill children: how family members navigate complex healthcare before, during and after hospitalisation in a rural Kenyan setting	Kenya [Child health]	Access to resources [Who has what?]	The lack of rapid access to money was an important contributing factor to a child's deteriorating condition; it influenced the initiation of a treatment-seeking. For instance, women made many references to "waiting to talk to my husband," "waiting to be sent money from my husband," and waiting for "his permission to pursue an action." Most primary caretakers, including mothers, were not income earners, and often relied heavily on their spouses or other household providers for money. At times, this social support was reported to be a burden and to cause delays in care-seeking or create an additional financial burden for households.
10	Storeng et al. (2012)	Too poor to live? A case study of vulnerability and maternal mortality in Burkina Faso	Burkina Faso [Maternal health]	Social norms [How are values defined?]	The study highlighted how structural impediments, including those related to motherhood and childbearing, limit individual resilience. For example, the social importance of bearing a child as soon as possible [amidst being physically or mentally capable] and the stigma associated with childlessness contributed to the death of the study actor who, despite having access to skilled birth attendance and emergency obstetric care, could not be rescued from maternal mortality.
11	Den Hollander et al. (2018)	Power difference and risk perception: mapping vulnerability within the decision process of pregnant women towards clinical trial participation in an urban middle-income setting	Ghana [Maternal health]	Agency and decision-making [Who decides?]	This study found a wide power difference between health providers and patients, and a different perception of risk through externalisation of responsibility of risk management within a religious context and a context shaped by authority. Therapeutic misconception was also observed, leading women to rely on the opinion of the medical professional, rather than being guided by their own motivation to participate. On their decision to participate in clinical trials, four women said that they would want to consult their husbands (or other family members living with them) before agreeing to participate. They

S/N	Author Year	Publication Title	Country [Context]	Main-Theme	Main Findings
					claimed that they would not be able to say they would participate without discussing it with the partner.
12	Arcos et al. (2011)	Vulnerability of pregnant women living in a community of metropolitan Santiago	Chile [Maternal health]	Access to resources [Who has what?]	It was observed that reproductive, neonatal, and mental and family health deteriorates when women have precarious jobs, with pregnancies without social security, informal and insecure habitability of housing, low schooling, psychosocial risk, absence of a partner in the home, and high dependency ratio.
13	Reyes et al. (2020)	Intimate partner violence and postpartum emotional distress among South African women: moderating effects of resilience and vulnerability factors	South Africa [Maternal health]	Power negotiation [How is power enacted, negotiated, or challenged?]	Consistent with the research hypothesis, this study found that the strength of the association between exposure to intimate partner violence and postpartum distress was stronger for women who reported greater levels of distress during pregnancy and weaker for women with greater socioeconomic assets. Intimate partner exposure impacts were greater for women reporting higher levels of emotional distress during pregnancy suggests that IPV-exposed women may be particularly likely to experience persistence (vs. discontinuity) of poor mental health across the antenatal and postnatal periods.
14	Warren et al. (2018)	"Sickness of shame": investigating challenges and resilience among women living with obstetric fistula in Kenya	Kenya [Maternal health]	Access to resources [Who has what?]	Most women affected by fistula had secondary education and a very low monthly income. It was reported that irrespective of marital status, having male support (e.g. husband, brother, or uncle), particularly financial support and help in securing transport to hospitals for repair care, was critical. This was seen especially in cases where facilities denied care to women who were unaccompanied by their husbands.
15	Lavender et al. (2020)	Journey of vulnerability: a mixed-methods study to understand intrapartum transfers in Tanzania and Zambia	Tanzania and Zambia [Maternal health]	Access to resources [Who has what?]	Women were usually not in control of their own finances, making them reliant on their partners for support; this made it difficult for them to be ready for emergency situations. Some women failed to attend clinics as a result of the lack of support from their husbands.

S/N	Author Year	Publication Title	Country [Context]	Main-Theme	Main Findings
					*Most husbands did not give their wives adequate funds for the needs during delivery.
16	Pourette (2012)	Haitian women living with HIV in Guadeloupe: pregnancy between medical issues, social benefits and administrative vulnerability: a qualitative study	Guadeloupe [Maternal health]	Access to resources [Who has what?]	In one interview with a respondent, the woman received financial support (e.g. rent fee, feeding) from her sexual partner.
17	Prates Cde et al. (2008)	Gender power, poverty and contraception: multiparous experiences	Brazil [Maternal health]	Agency and decision-making [Who decides?]	The authors illuminated the challenges of multiparity in poorest sections of the population, where precarious living conditions, including inequalities in gender power, resulted in reduced potential empowerment of women to organise their lives, including planning childbirth. *Male resistance to condom use appeared to be prominent in the testimonials. The decrease in this autonomy affected the reproductive life, reducing its power to control one’s fertility, which resulted in multiparity.
